# Evaluation of d 18 in ovo administration of soursop (Annona muricata) leaf extract into the air space on hatch performance and physiology of Noiler chicks

**DOI:** 10.1016/j.psj.2024.104220

**Published:** 2024-08-22

**Authors:** Timothy T. Kuka, Batomayena Bakoma, Jacob Hamidu, Okanlawon Onagbesan, Kokou Tona

**Affiliations:** ⁎ Regional Center of Excellence in Poultry Science, University of Lome, P.B. 1515, Lome, Togo; † Pharmaceutical Sciences Research Laboratory, Department of Pharmacy, University of Lome, P.B. 1515, Lome, Togo; ‡ Department of Animal Science, Kwame Nkrumah University of Science and Technology, P. O. Box Up 1279, Kumasi, Ghana; § Department of Animal Nutrition, Joseph Sarwuan Tarka University, P.M.B. 2373, Makurdi, Nigeria

**Keywords:** hatch performance, in ovo, Noiler, soursop, leaf extract

## Abstract

Efficient poultry production can be accomplished using combined technologies. A combination of in ovo and ethno-veterinary technologies can deliver significant benefits, including reduced labour and production costs. This study evaluated the effect of in ovo administration of soursop leaf extract (**SLE**) on the hatching performance and physiology of Noiler chicks. A total of 550 eggs were incubated, and 460 fertile eggs were randomly distributed into 4 groups with 3 replicates on the 18th d of incubation. The eggs were then injected with 0.75 µg SLE, 1.5 µg SLE, and saline solution (negative control) at a rate of 0.2 ml in the air space. The noninjected group served as the positive control. At the end of the hatching period, the various groups were evaluated for embryo mortality, hatchability, hatch duration, organ weight, serum biochemistry, and chick quality. The results showed no significant differences (*P* > 0.05) in embryonic mortality, hatchability, organ weight, total protein, albumin, globulin, aspartate aminotransferase, alanine aminotransferase, and glucose among the treatment groups. However, chick weight, chick quality, and serum triglyceride levels were significantly (*P* < 0.05) higher in the extract-injected group. Additionally, incubation and hatch times were significantly lower (*P* < 0.05) in the SLE group compared to the other groups. In ovo administration of soursop leaf extract resulted in reduced incubation duration, hatch time, and embryo mortality. In conclusion, the in ovo injection of SLE improved hatch performance and chick quality.

## INTRODUCTION

Exploration of natural substances as an alternative strategy to enhance poultry health and productivity, while reducing the reliance on conventional medications and antibiotics has become necessary due to legislation, consumer demand and implications on the cost of production (Valenzuela-Grijalva et al., 2017; Puvača et al., 2020). Among these alternatives, botanical extracts have gained attention because of their potential to improve various aspects of poultry production, including growth performance, immune function, and disease resistance (Hotea et al., 2023). Noiler chicken is an indigenous dual-purpose breed that is gaining recognition for its hardiness, adaptability, and efficient meat and egg production traits, particularly in smallholder farming systems in West Africa (Noiler.net, 2024). Its introduction has changed the indigenous poultry industry by increasing the number of eggs produced per season, increasing the meat yield and shortening the rearing cycle for meat production, which has encouraged more farmers to adopt it for commercial farming.

However, maintaining optimal health and performance remains crucial for the sustainability of Noiler chicken production and the poultry industry. Soursop (*Annona muricata*), a plant native to tropical regions is one such botanical source that has attracted significant interest. Soursop leaves have a long history of traditional use in folk medicine for their health benefits, ranging from antimicrobial and anti-inflammatory properties to potential anticancer effects (Pai et al., 2016; Mutakin et al., 2022). Within the context of poultry production, studies have explored the potential of soursop leaf extract as a natural supplement to promote growth performance, enhance immunity, and mitigate the negative impacts of stressors on poultry production.

Oluwayinka et al. (2017) reported that the administration of a 200mg/kg dosage of an aqueous extract of *Annona muricata* reduced the immune response to Newcastle disease vaccine in broiler chickens, without any negative effects on their haematological and liver functions. Similarly, Jiwuba et al. (2017) found that oral administration of soursop leaf extract, at a concentration of 40 ml/liter in drinking water for broiler chickens, as an alternative growth promoter improved the blood profile of the chickens. Furthermore, the oral administration of soursop leaf extract in broiler chickens positively influenced the feed conversion ratio and intestinal morphology and reduced intestinal microbial colonization at doses ranging from 7.5% to 12.5% (Kuka et al., 2022). Artawiguna et al. (2023) also reported that administering fermented soursop (Annona muricata) leaf extract at a concentration of 2% through drinking water improved performance variables and microbes in the digestive tract of Joper male chickens.

Unfortunately, oral administration can only be applied during the rearing of the chicks thereby omitting the perinatal period within which important physiological activities such as intestinal development and colonization happen. Furthermore, more resources (quantity of the extract) may be required for oral administration, hence the consideration of in ovo injection as a route of administration. The in ovo technique, which involves the administration of substances directly into the egg during incubation, is a novel approach for delivering bioactive compounds to the developing embryos to improve their performance after hatching (Pawłowska et al., 2022; Kpodo and Proszkowiec-Weglarz, 2023). Administration of soursop leaf extract via in ovo injection offers a unique avenue for potentially enhancing the hatching processes and overall health of Noiler chicken embryos and the chicks. The biological activities of soursop leaf extract, including antioxidant, antimicrobial, and immunomodulatory properties, hold assurance for positively influencing embryonic physiology and subsequent hatchling performance (Abdul-Wahab et al., 2018). The approach of directly targeting the embryonic stage may exert profound effects on chick weight, hatchability, chick quality, mortality and post-hatch performance of the chicks.

This study is a follow-up to our previous work (Kuka et al., 2023), where foundational levels (0.00, 0.25, 0.5, and 0.75 µg) of soursop leaf extract were administered in ovo. The influence on the hatching and posthatch performances of Noiler chicks was dose-dependent; the lowest concentration negatively affected the performance while the other concentrations showed mixed outcomes. Our desire to validate the effect of in ovo administration of soursop leaf extract on the performance of Noiler chickens through a comprehensive assessment of hatching events, hatchability, embryonic mortality, chick quality, organ weights, and serum biochemistry led to the choice of the present doses. This understanding will not only contribute to the optimum use of soursop leaf extract to improve performance but also align with the growing demand for sustainable and natural alternatives in poultry production.

## MATERIALS AND METHODS

The present study was conducted at the hatchery section of the experimental unit of the Regional Center of Excellence in Poultry Science (CERSA), following the approval (008/2021/BC-BPA/FDS-UL) by the research and development committee, University of Lome, Togo.

### Preparation of the Leaf Extract

Soursop leaves were harvested within the maritime region of Togo. The leaves were air-dried under air condition at 20°C and milled into powder for use. Leaf extraction followed a modification of the procedure described by Cheng et al. (2021). Five hundred (500) g of soursop leaf powder was macerated in 5 liters of ethanol (80%). The mixture was continuously agitated for 72 h, after which it was filtered through a 2 cm layer of cotton wool laid in a filtering funnel and finally through a coffee filter (# 6) fitted in a filtering funnel. The filtrate was concentrated using a rotary evaporator with a chiller and vacuum pump at 40°C to obtain the concentrated leaf extract that was refrigerated for reconstitution and use. Ten (10) mg of the extract were reconstituted with 100 mL of saline solution (0.9%) to obtain the stock solution (1000 µg). One ml of the stock solution was again reconstituted with 100 ml of saline solution to produce a 10 µg working solution. Finally, 7.5 ml and 15.0 mL of the working solution were reconstituted with 10 mL of saline solution, respectively, to obtain 0.75 and 1.5 µg concentrations to be used for the injection.

### Experimental Design and Injection

A completely randomized design was used for this experiment. Five hundred and fifty (550) hatching eggs of Noiler chicken were obtained from a breeder stock of 42 wk old in Lome. The eggs were weighed, numbered, and incubated at a temperature of 37.7°C and relative humidity of 60% in a Royal Pas Reform (SmartPro) combi incubator, Netherlands. The incubator was set to automatically turn the egg at an hour interval and an angle of 45^o^. On the 18th d of incubation, eggs were candled to remove infertile ones and eggs with evidence of living embryos were randomly assigned to 4 groups of 115 eggs each. Group 1 eggs were neither perforated nor injected, group 2 eggs were injected with saline solution, groups 3 and 4 were injected with 0.75 µg and 1.5 µg of soursop leaf extract. All the eggs were replicated trice in hatching baskets and placed in the hatching machine (Royal Pas Reform), where the temperature and relative humidity were adjusted to 37.5°C and 70 % respectively, and the eggs were observed for hatching according to their groups. Before the injection, the eggs were disinfected with cotton wool dipped in alcohol (80%). Two holes were made on the broad side of the eggs using 21 g needles to facilitate the delivery of the extract. The extract was injected using an automatic syringe with a 22 g (13 mm) needle, and the holes were sealed with adhesive tape. All the injected eggs received 0.2 ml of saline and leaf extract in the air space. The eggs were then observed for hatching activities (internal, external pipping and emergence) and records were taken at 3-h intervals beginning from the 444th h.

#### Data and Sample Collection

***Hatching Events and Chick Quality***: From 444th h of incubation, internal pipping (**IP**), external pipping (**EP**), and chick emergence for each egg were recorded. Eggs were considered internally pipped by the appearance of the chick's beak in the air space or through the inner membrane of the egg when viewed under the light while external pipping was recognized by the crack in the eggshell and appearance of the beak.

The data collected was used to evaluate cumulative internal, external pipping, internal pipping duration (time of external pipping - time of internal pipping), external pipping duration (time of hatch - time of external pipping), hatch duration (time of hatch-time of internal pipping), hatch window (hatch time of the last chick – hatch time of the first chick) and spread.

The percentage hatchability was calculated using the formula below:

$$Hatchability\;\left(\%\right)=\frac{Number\;of\;hatched\;chicks}{Number\;of\;fertile\;eggs}\times 100$$

The unhatched eggs were broken to ascertain their state and determine embryo mortality which was calculated as follows:

$$Mortality\;\left(\%\right)=\frac{Number\;of\;dead\;chicks}{Number\;of\;fertile\;eggs}\times 100$$

Early mortality was considered as those that occurred before d 18 (not fully developed) while late mortality was considered from d 18 of incubation and above. At the end of the hatch, eighty (80) of the hatched chicks from each group were weighed and assessed for chick quality based on physical observations such as activity, the eyes, navel area, legs, retracted yolk, remaining membrane, remaining yolk, down and appearance using the Tona score presented in Table 1 (Tona et al., 2003). The quality of a chick was defined as the sum of scores for each parameter in line with the characteristics.

**Table 1:** Tona score chart.

Parameters	Characteristics	Scores
Activity	Good Dull	6 0
Down and appearance	Dry	10
	Wet	8
	Dirty and wet	0
Retracted yolk	Normal abdomen	12
	Enlarged and hard	0
Eyes	Open and bright	16
	Open and dull	8
	Closed	0
Legs	Normal legs and toes	16
	One leg affected	8
	Both legs affected	0
Navel	Completely closed and clean	12
	Not completely closed and not discoloured	8
	Not closed but discoloured	0
Remaining membrane	No membrane	16
	Little membrane	8
	Large membrane	4
	Very large membrane	0
Remaining yolk	No yolk	16
	Little yolk	8
	Large yolk	4
	Very large yolk	0
Total	Sum of scores per chick	100–0

Tona et al., 2003.

***Blood and organ collection***: Two chicks per replicate were weighed and sacrificed by severing the jugular vein. Blood samples were collected into dry tubes, allowed to clot, and centrifuged at 3000 rpm for 15 min to harvest serum. Total protein, albumin, globulin, alanine aminotransferase (**ALT**), aspartate aminotransferase (**AST**) and triglycerides in the serum were determined using specific reagents (Cypress Diagnostic, Belgium) and Gen5 Microplate Reader and Imager Software (BioTek Instruments), Following manufacturer's instruction. Blood glucose levels were determined immediately at the hatchery using an Accu-Check glucometer with strips.

Hatch muscles, heart, liver, yolk sac, lungs and the complete gastrointestinal tract, were harvested and weighed using a sensitive balance. The weights of these organs were expressed as a percentage of the body weight using the formula below to obtain the relative organ weights.

$$Relative\;weight=\frac{Weight\;of\;organ}{Weight\;of\;chick}\times 100$$

### Statistical Analysis

The data collected were computed using percentages and 1-way analysis of variance (ANOVA) in SPSS Software (Version 23). Differences between treatment means were compared using Tukey's honest significant difference test at *P* ≤ 0.05. The following model was used: Yij = µ + Ti + Eij; where µ = the population mean; Ti = the effect of each in ovo injection treatment and Eij = the residual error. Graph pad prism software was used to plot the charts.

## RESULTS AND DISCUSSION

### Hatch Performance

**Figure 1:**
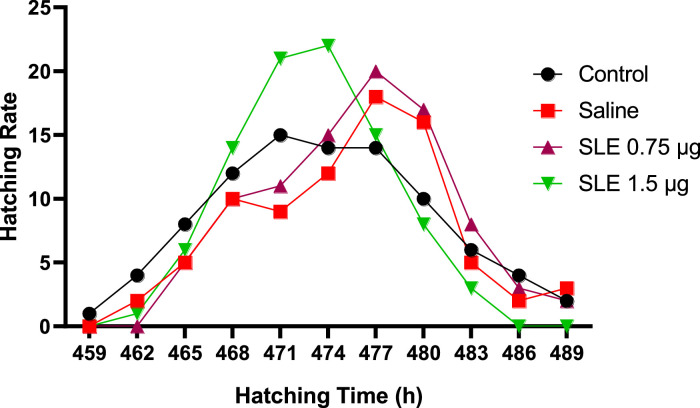
Effect of in ovo soursop leaf extract on emergence of Noiler chicks.

The effect of in ovo administration of soursop leaf extract (SLE) on the hatching activities of Noiler chicks is presented in Table 2. Significant differences (*P* < 0.05) were observed in the internal pipping, external pipping, and emergence of the chicks and the total incubation time of the eggs. A significantly shorter incubation and hatching time was observed for the Soursop leaf extract at 1.5 µg compared to the control and saline groups (*P* = 0.024; *P* = 0.016; *P* =0.012). Internal and external pipping time was shorter in the SLE at 1.5 µg compared to the other groups (Table 2 and Figure 1). Soursop leaf extract at 1.5 µg positively influenced the hatching process, thereby reducing the incubation time and hatching process. In contrast, the control group had the longest incubation and hatching time. This can be attributed to the bioactive composition of the leaf extract whose effect in this regard can best be described as synergistic, leading to reduced oxidative stress, enhanced embryo vitality and subsequent hatching performance. Abdul-Wahab et al. (2018) affirmed that the biological activities of soursop leaf extract, which includes antioxidant, antimicrobial, and immunomodulatory properties, hold assurance for positively influencing embryonic development. The results of this study are consistent with those of Ngueda et al. (2021) who also observed reduced pipping and incubation time of Sasso eggs with in ovo injection of *Manihot esculenta* leaf extract and Bilalissi et al. (2019) who observed reduced incubation time with in ovo administration of *Moringa oleifera* leaf extract.

**Table 2:** Effect of d 18 *in ovo* administration of soursop leaf extract in the air space on incubation and hatching activities of Noiler chicken eggs.

			Treatments (SLE)			
Parameters	Control	Saline	0.75 µg	1.5 µg	SEM	*P*-value
IP – duration (h)	12.86^a^	12.15^a^	12.33^a^	11.86^b^	0.172	0.024
EP – duration (h)	14.05^a^	12.25^a^	11.25^b^	7.42^c^	0.428	0.016
Hatch window (h)	30.01^a^	27.00^a^	24.18^b^	21.13^c^	0.902	0.009
Total hatch time (h)	45.00^a^	45.00^a^	42.00^b^	39.00^c^	0.488	0.032
Total incubation time (h)	489.55^a^	489.25^a^	489.00^a^	483.50^b^	0.281	0.012

Abbreviations: EP, external pipping; IP, internal pipping; SLE, soursop leaf extract.

n = 115.

a,b,c Mean within a row with different superscripts are significantly different.

The effect of in ovo administration of soursop leaf extract on the hatching rate and time of Noiler chicks is shown in Figure 1. Chicks from the control group emerged first beginning at the 15th h after internal pipping and attained their peak at the 27th h but ended up last taking a total of 45 h. The Saline, 0.75 and 1.5 µg extract groups began emerging at the 18th h after internal pipping, the 1.5 µg group took 39 h to complete the hatching process reaching their peak at the 30th h whereas the saline and 0.75 groups attained their peak hatch at 33^rd^ h and ended hatching process at 45th h after internal pipping. The hatch window for the 1.5 group was 21 h followed by saline (27 h) and 0.75 group (27 h) and the control group (30 h). Soursop leaf extract positively influenced the hatching process by reducing the total incubation time by 6 h compared to the other groups. This reduction in incubation time can save energy and space for hatchery managers, thereby optimizing their operations. It should be noted that the incubation time was counted from when incubator parameters (temperature and humidity) were set after the incubator door was closed to when the last chick was hatched.

Table 3 shows the effect of in ovo administration of soursop leaf extract on hatchability and embryo mortality of Noiler chicks. No significant effect (*P* > 0.05) was observed on the percentage hatchability of fertile eggs as well as total embryo mortality. However, injection of saline solution resulted in reduced hatchability while soursop leaf extract increased hatchability numerically (not statistically). Total embryo mortality was also numerically highest in the saline-injected group but lowest in the group administered the highest concentration of soursop (1.5 µg). The antioxidant and antibiotic properties of soursop leaf extract can significantly contribute to the survival of chicken embryos if a sufficient amount is administered.

**Table 3:** Effect of d 18 *in ovo* administration of soursop leaf extract in the air space on hatchability and embryo mortality of Noiler chickens.

			Treatment (SLE)			
Parameter	Control	Saline	0.75 µg	1.5 µg	SEM	*P*-value
Hatchability %	78.21	71.26	79.10	78.25	1.429	0.172
Total unhatched	8.33	11.00	8.00	8.33	0.529	0.141
Early mortality	5.00	5.67	5.33	4.00	0.275	0.150
Contaminated eggs	3.00	3.67	1.67	2.33	0.333	0.163
Alive but unhatched	0.33	0.00	0.33	0.33	0.131	0.802
Late mortality	0.00	1.67	0.67	1.67	0.326	0.193
Total embryo mort %	13.07	14.78	13.90	10.46	0.722	0.164

Abbreviations: *P*-value, probability; SEM, standard error of the mean; SLE, soursop leaf extract.

Late mortality refers to the fully formed chicks including those that pipped externally but died.

n = 115.

The result indicated that the leaf extract was not detrimental to the embryo and that the injection process did not negatively affect the embryos. This observation was consistent with our previous study where lower concentrations of soursop leaf extract were used in Noiler chickens but no significant difference was observed in the hatchability and embryo mortality (Kuka et al., 2023). Kop-Bozbay and Göneci (2023) reported a similar result with Cinnamon, ginger and Anise extract in broiler chicken. Ngueda et al. (2021) also reported similarity in hatchability and mortality rate with in ovo injection of cassava leaf extract in Sasso chicken eggs. Similar results have been reported for in ovo administration of Pollen, propolis and curcumin extracts, which have identical attributes (Coskun et al., 2014; Aygun 2016; Heidary *et al.*, 2020).

### Chick Quality

Table 4 shows the effect of in ovo administration of soursop leaf extract on chick quality of Noiler chickens. Significant differences were observed in chick weight, down and appearance, remaining membrane, and overall quality scores between the groups. These parameters were significantly improved by in ovo administration of soursop leaf extract.

**Table 4:** Effect of d 18 *in ovo* administration of soursop leaf extract in the air space on quality of Noiler chicks.

			Treatment (SLE)			
Parameter	Control	Saline	0.75 µg	1.5 µg	SEM	*P*-value
Chick weight (g)	36.99^b^	37.73^b^	38.62^b^	40.71^a^	0.275	<0.001
Activity	6.00	6.00	6.00	6.00	0.000	-
Down and appearance	9.71^b^	9.80^b^	9.93^a^	9.93^a^	0.028	0.011
Retracted yolk	11.98	12.00	12.00	12.00	0.007	0.393
Eyes	15.90	15.40	16.00	16.00	0.025	0.393
Legs	16.00	16.00	16.00	16.00	0.000	-
Naval area	11.67	11.61	11.66	11.76	0.078	0.936
Remaining membrane	11.82^b^	11.90^b^	11.97^a^	12.00^a^	0.050	0.001
Remaining yolk	14.36	14.64	15.08	15.63	0.105	0.172
Total quality score	96.00^b^	96.07^b^	98.57^a^	99.22^a^	0.209	<0.001

Abbreviations: *P*-value, probability; SEM, standard error of the mean; SLE, soursop leaf extract.

n = 80.

a,b,c Mean within a row with different superscripts are significantly different.

The effect of SLE on chick weight and quality scores was observed to be dependent on the concentration of the extract. Bioactive substances such as β-carotenes, ascorbic acid, flavonoids, saponins, alkaloids, tannins and glycosides (Agu and Okolie, 2017) present in soursop leaf extract provide antioxidative and antimicrobial properties that are believed to have influenced embryo health and proper naval healing leading to active and clean chicks.

The findings of this study agree with those of Ngueda et al. (2021), who observed a significant increase in the chick weight and overall quality scores of Sasso chicks administered in ovo cassava leaf extract. Kop-Bozbay and Göneci (2023) found that in ovo administration of cinnamon, ginger and Anise extracts significantly increased the weight of broiler chicks. N'nanle et al. (2017) also reported increased chick weight while Bilalissi et al. (2019) noticed increased chick quality with the administration of in ovo moringa leaf extract.

### Relative Organ Weights

Table 5 shows the effect of in ovo soursop leaf extract on the relative organ weights of Noiler chicks. No significant difference was observed in the liver, heart, lungs, GIT, yolk sac and hatch muscles. Nonetheless, hatch muscle and yolk sac were numerically higher in extract-administered groups than in the other groups. The yolk serves as a source of nutrients for the developing embryo and provides nutrients for the newly hatched chicks pending when it will be fed (Sahan et al., 2014). A bigger yolk sac contributes to the weight of the chick and provide more nutrients for the chick to survive during the starvation periods. The hatching muscle is a vital organ that assists the egg tooth to bring about the hatching of the chicks. Embryos with less developed hatch muscles are likely to die in the shell after pipping (Harvey, 1958); thus, higher hatching muscles lead to higher hatch rates.

**Table 5:** Effect of d 18 *in ovo* administration of soursop leaf extract in the air space on vital organs of Noiler chicks at hatch.

	Treatments (SLE)					
Parameter (%)	Control	Saline	0.75 µg	1.5 µg	SEM	*P*-value
Hatch muscle	0.99	0.97	1.07	1.02	0.059	0.567
Heart	0.70	0.73	0.67	0.64	0.018	0.131
Liver	2.62	2.80	2.52	2.59	0.059	0.151
Lungs	0.77	0.70	0.73	0.70	0.033	0.495
GIT	11.33	10.67	8.79	10.26	0.430	0.058
Gizzard	6.79	6.19	5.33	6.10	0.257	0.070
Yolk residue	10.45	11.37	15.93	12.68	1.036	0.092

Abbreviations: GIT, gastrointestinal tract; P-value, probability; SEM, standard error of mean; SLE, soursop leaf extract.

n = 6.

The results of this study are similar to those of Oke et al. (2021), who reported similarity in the liver, yolk sac, lungs, intestine and gizzard weights of broiler chicks injected with black cumin extract. Kop-Bozbay and Göneci (2023) also reported similarities in the yolk sac, heart, liver, gizzard and intestinal weights of broiler chicks injected with Cinnamon, Ginger and Anise extracts in ovo. In contrast, Bilalissi et al. (2019) reported increased liver and yolk sac but similar heart weight when they administered in ovo moringa leaf extract. In another study, Ngueda et al. (2021) reported increased heart, hatch muscles and liver weights but reduced yolk sac with the *in ovo* administration of cassava leaf extract. On the other hand, N'nanle et al. (2017) found a similarity in liver and heart weights with a reduced hatch muscle in Sasso chicks. The similarities in organ weights in this study indicated that soursop leaf extract administration was not harmful to the organs.

### Serum Biochemistry

The effect of in ovo soursop leaf extract on serum biochemistry of Noiler chicks at hatch is presented in Table 6. No significant difference was observed in the total protein, albumin, globulin, aspartate aminotransferase, alanine aminotransferase, and glucose. Serum proteins were more similar in extract-injected groups than the others while blood glucose was higher in extract-injected groups than in the other groups. Triglycerides were significantly higher (*P* < 0.05) in the injected groups than in the noninjected groups. Serum parameters are indicators of the health status of an animal; they reveal toxicity, inflammation and adverse conditions affecting the animal. Their similarities in this study indicated the safety of the extract used. Glucose and triglycerides indicate the energy level of an animal's body, the increased triglycerides values in the extract-injected groups suggest that soursop leaf extract must have exhibited nutritional benefit that led to higher energy reserve in the chicks as can also be observed in the numerically higher yolk sac. Ngueda et al. (2021) reported an increase in triglycerides with a decrease in AST and ALT in Sasso chicks injected with cassava leaf extract. Oke et al. (2021) also reported a decrease in AST and ALT but did not observe a difference in total proteins, albumin, globulin, triglycerides and glucose with in ovo administration of black cumin in broiler chicks. Higher triglycerides indicate the availability of energy for hatching activities, which prevents the chicks from depleting the muscle reserve as well as consuming the yolk sac, which normally leads to stress and less weight at the hatch. Chicks with high triglycerides and glucose levels can perform better even if there are delays in post-hatch feeding; this is evident in this study, as chicks with higher triglycerides value (extract-injected) had better hatch, less embryo mortality and higher weights.

**Table 6:** Effect of d 18 *in ovo* administration of soursop leaf extract in the air space on serum biochemistry of Noiler chicks at hatch.

			Treatments (SLE)			
Parameter	Control	Saline	0.75 µg	1.5 µg	SEM	*P*-value
Total protein (g/dL)	4.22	4.84	4.54	4.57	0.089	0.094
Albumin (g/dL)	2.45	2.43	2.61	2.65	0.048	0.258
Globulin (g/dL)	1.77	2.42	1.94	1.92	0.093	0.065
ALT (U/L)	14.73	21.07	22.03	17.66	1.172	0.099
AST (U/L)	24.17	43.09	45.22	36.64	3.428	0.119
Triglycerides (mg/dL)	163.56^b^	223.31^a^	226.95^a^	247.80^a^	9.902	0.009
Glucose (mg/dL)	168.83	170.00	168.33	170.50	4.188	0.082

Abbreviations: ALT, alanine aminotransferase; AST, aspartate aminotransferase; SLE, soursop leaf extract.

n = 6.

a,b Mean within a row with different superscripts are significantly different.

## CONCLUSION

The study evaluated the influence of the 18th d in ovo administration of soursop leaf extract into the air space on hatch performance and physiology of Noiler chicks. Soursop leaf extract enhanced the incubation and hatching process by reducing the hatch window by 6 h and the entire incubation accomplished in 483.5 h against 489.55 h. In ovo administration of soursop leaf extract also improved chicks’ weight, quality and serum energy levels. However, in ovo administration of soursop leaf extract could not significantly improve embryo mortality, yolk sac, hatching muscles, and blood glucose of the hatched chicks. In all, in ovo soursop leaf extract enhanced the hatching process as well as the weight and quality of Noiler chicken without detrimental effects. The effect of the extract was dose-dependent; hence, higher concentrations of the extract should be explored for optimum benefits.

### Declaration of AI-Assisted Technologies in the Writing Process

During the preparation of this work, the author(s) used EditGpt to improve grammar in some sections. After using this tool/service, the author(s) reviewed and edited the content as needed and take(s) full responsibility for the content of the publication

## DISCLOSURES

The authors declare that there are no competing interests with this work.
